# Species‐specific adaptations explain resilience of herbaceous understorey to increased precipitation variability in a Mediterranean oak woodland

**DOI:** 10.1002/ece3.1662

**Published:** 2015-09-09

**Authors:** Marjan Jongen, Christine Hellmann, Stephan Unger

**Affiliations:** ^1^ Instituto Superior de AgronomiaUniversidade de LisboaTapada da Ajuda1349‐017LisboaPortugal; ^2^ Department of Experimental and Systems EcologyUniversity of BielefeldUniversitätsstr. 25D‐33615BielefeldGermany; ^3^ AgroEcosystem ResearchBayCEERUniversity of BayreuthUniversitätsstr. 30D‐95447BayreuthGermany

**Keywords:** *Agrostis pourretii*, climate change, Mediterranean ecosystem, *Ornithopus sativus*, precipitation manipulation, *Rumex acetosella*, *Tolpis barbata*, *Tuberaria guttata*

## Abstract

To date, the implications of the predicted greater intra‐annual variability and extremes in precipitation on ecosystem functioning have received little attention. This study presents results on leaf‐level physiological responses of five species covering the functional groups grasses, forbs, and legumes in the understorey of a Mediterranean oak woodland, with increasing precipitation variability, without altering total annual precipitation inputs. Although extending the dry period between precipitation events from 3 to 6 weeks led to increased soil moisture deficit, overall treatment effects on photosynthetic performance were not observed in the studied species. This resilience to prolonged water stress was explained by different physiological and morphological strategies to withstand periods below the wilting point, that is, isohydric behavior in *Agrostis*,* Rumex,* and *Tuberaria*, leaf succulence in *Rumex*, and taproots in *Tolpis*. In addition, quick recovery upon irrigation events and species‐specific adaptations of water‐use efficiency with longer dry periods and larger precipitation events contributed to the observed resilience in productivity of the annual plant community. Although none of the species exhibited a change in cover with increasing precipitation variability, leaf physiology of the legume *Ornithopus* exhibited signs of sensitivity to moisture deficit, which may have implications for the agricultural practice of seeding legume‐rich mixtures in Mediterranean grassland‐type systems. This highlights the need for long‐term precipitation manipulation experiments to capture possible directional changes in species composition and seed bank development, which can subsequently affect ecosystem state and functioning.

## Introduction

The Mediterranean climate in the Iberian Peninsula is characterized by relatively mild and wet winters and hot and dry summers, with high temperatures and low soil moisture in the June to September period setting the abiotic limit for productivity (e.g., Tenhunen et al. 1990). The understorey vegetation in the savanna‐type evergreen oak woodlands is dominated by C3 annual plant species, which avoid the dry hot summer period by adjusting their life cycle to the seasonal water availability (e.g., Unger et al. 2009). Nevertheless, the vegetative and reproductive growth during the life cycle of these annual species exhibits a strong dependence on water availability.

For the Mediterranean region, climate change scenarios predict decreasing annual precipitation (Christensen et al. 2007), accompanied by changes in seasonality and temporal variability of precipitation (Luterbacher et al. 2006), with a decrease in the number of precipitation days and an increase in the length of the dry spells (Easterling et al. 2000).

Indeed, studies report an increase in the dry period between precipitation events in the Iberian Peninsula (Gallego et al. 2011), with increasing drought frequency in Portugal for the February to March period (Pires 2003). The increase in precipitation variability is expected to extend the periods of soil moisture deficit (Jackson et al. 2001), which might have important consequences for productivity, biodiversity, and the matter cycles of many terrestrial ecosystems (Austin et al. 2004; Huxman et al. 2004; Schwinning and Sala 2004), and particularly for drought‐susceptive grasslands (Knapp et al. 2002; Harper et al. 2005; Fay et al. 2011).

To date, several studies have reported on the effects of alterations in the precipitation regime (without changing total precipitation inputs) on primary productivity and species composition. In arid ecosystems, no significant changes in productivity with increasing precipitation variability were found (Miranda et al. 2009; Thomey et al. 2011; Vargas et al. 2012), although Thomey et al. (2011) did report significant productivity increases in the dominant grass species in plots receiving large monthly rainfall events, as compared to plots receiving small weekly rainfall events. In marked contrast, in semi‐arid ecosystems positive effects of larger infrequent precipitation events on productivity were found (Heisler‐White et al. 2008, 2009), whereas negative effects on productivity have been reported for mesic ecosystems (Knapp et al. 2002; Laporte et al. 2002; Fay et al. 2003; Harper et al. 2005; Heisler‐White et al. 2009). However, the majority of studies, reporting on effects of increased precipitation variability in grassland ecosystems, report no significant changes in productivity or species composition (Unger and Jongen 2015).

To develop a better understanding of the effects of altered precipitation regimes on ecosystem processes in the herbaceous understorey in a Mediterranean oak woodland, this understorey vegetation resembling Mediterranean grasslands, we established a large‐scale rainfall manipulation experiment. The aim was to assess the impact of increasing precipitation variability, without altering total annual precipitation inputs, on productivity and species composition of the understorey vegetation. In previous studies, we found no significant effects on net primary productivity and community structure when extending the dry period between precipitation events from 1 to 3 weeks (Jongen et al. 2013a). The absence of differences in productivity with precipitation variability in the latter study was explained by the apparent lack of severity in drought stress caused by the changing precipitation patterns. Extending the dry period to 6 weeks in a consecutive study was expected to challenge the phenotypic and physiological plasticity of understorey vegetation, with potentially severe consequences for productivity and carbon sequestration (Jongen et al. 2013b). However, although in this study increased soil moisture deficit with 6‐weekly precipitation frequency was reported, no effects on productivity and community structure were found (Jongen et al. 2013b), with the herbaceous understorey being highly resilient to increased precipitation variability. This finding suggests employment of stress avoidance and/or adaptation strategies of the understorey vegetation when exposed to moisture stress, with changes in morphological, physiological, and biochemical mechanisms. For example, plants can adjust water‐use efficiency, by minimizing water loss, while maintaining photosynthesis, primarily achieved by decreasing stomata aperture, increasing photosynthetic capacity, or both (Bacon 2004; Buckley 2005; Gilbert et al. 2011). Alternatively, plants can avoid stress by rapidly completing their life cycle before the onset of severe drought (Geber and Dawson 1997; Chaves et al. 2003), develop extensive root systems, and increase root to shoot ratio to enhance water uptake in relation to water loss (Comas et al. 2013; Lynch 2013), increase structural carbon to withstand turgor losses with decreasing leaf water potentials (Brugnoli et al. 1998; Monti et al. 2006; Werner and Máguas 2010), or use strategies to protect against photo‐inhibition (Werner et al. 2002; Galmés et al. 2007). The ability of the plant community to cope with a temporary soil moisture deficit results from the employment of a combination of strategies, probably associated with species‐specific traits (Valladares and Sánchez‐Gómez 2006; Berger and Ludwig 2014), with studies revealing a diversity of adaptive mechanisms among coexisting species (Pérez‐Ramos et al. 2013).

The present study attempts to describe temporal alterations in species physiology, resulting from altered precipitation regimes, with the length of the dry period between precipitation events extended to 6 weeks. We studied three prominent forb species, the dominant grass, and a legume, these species all co‐occurring in the herbaceous understorey of Mediterranean evergreen oak woodlands. We elaborate on the physiological adaptations of these species in periods of water deficit, and on the ability to recover with irrigation, in order to identify strategies, on a species‐ and functional group‐specific level, explaining the previously observed resilience to increased precipitation variability.

We hypothesized that (1) the resilience observed with increasing precipitation variability can be explained by species‐specific traits and water stress avoidance and/or adaptation strategies; (2) the different functional groups will have distinct strategies, with (3) the legume being least adapted to the increase in the extent of the dry period between precipitation events, with previous results showing increased sensitivity of this functional group to changing precipitation patterns. In addition, we hypothesize (4) that, upon irrigation, the ability of the studied species to recover is dependent on phenology, specifically whether senescence has been initiated.

## Materials and Methods

### Site description

The study was conducted at the Herdade da Machoqueira do Grou (39°08′16″N, 8°20′03″W), 30 km northeast of Coruche, Portugal. The soil is a Cambisol (FAO 2006), with 81% sand, 14% silt, and 5% clay. The ecosystem is limited in nitrogen, with total soil inorganic N at the experimental site during the study period being less than 1 *μ*g g^−1^ soil (Jongen et al. 2013b). Volumetric soil water content (*θ*_v_) at field capacity and at permanent wilting point is 19.3% and 7.6%, respectively. The climate is Mediterranean, characterized by wet and mild winters, and dry and hot summers. Long‐term mean annual temperature is ~15.9°C, and mean annual precipitation is 680 ± 210 mm (*Inst. de Meteorologia*, Lisbon), with 87% (594 mm) of the precipitation being confined to the growing season (October 1 to May 31).

The study site is located in a montado, a Mediterranean evergreen oak woodland, with *Quercus suber* being the only tree species. The tree density is ~45 trees ha^−1^. The understorey vegetation consists of a mixture of C3 annual species (e.g., *Agrostis pourretii, Tolpis barbata*,* Tuberaria guttata*,* Vulpia bromoides*), emerging after the first rains in autumn and senescing in late spring. Until October 2009, the experimental site was intermittently grazed with a stocking density of 0.16 cattle ha^−1^. In October 2010, the site was plowed and seeded with a legume‐rich mixture (a.o. *Ornithopus sativus*) and the grass *Lolium multiflorum* (seed mixture Charneca 650, S 07874, Fertiprado, Vaiamonte, Portugal). The species included in the mixture are typically native to Portugal or the Mediterranean region. Sowing legume‐rich seed mixtures in agro‐silvo‐pastoral systems, in order to improve productivity and soil fertility, is a common agricultural practice in Portugal (Crespo 2010).

### Experimental design and rainfall manipulation

In December 2009, eight rainfall manipulation shelters (greenhouse model “Fraga,” Prilux, Ponte de Vagos, Portugal) were constructed within a fenced area of ~3500 m^2^, enabling manipulation of the precipitation received by the understorey. Each shelter covered an area of 6 × 5 m (30 m^2^), with an eave height of 1.6 m and a ridge height of 2.5 m. The shelter roofs were covered in November 2011 by a clear, 2‐mm, UV‐transparent polyethylene glasshouse film (Plásticos F. Matos, Massamá, Portugal). For additional information on shelter design, see Jongen et al. (2013a). The two water manipulation treatments were as follows: “3‐weekly watering treatment” (3W), with a dry period between precipitation events of 21 days, and “6‐weekly watering treatment” (6W), with this dry period increased twofold to 42 days. Each of the two treatments had four replicate experimental plots. To prevent a treatment effect on germination and seedling establishment, all experimental plots were subjected to equal water inputs until the middle of November 2011, receiving 244 mm of natural precipitation. Subsequently, from November 17 onwards, when the shelters were covered, until the end of May, experimental plots were subjected to the water manipulation treatments, with 3W receiving 40 mm of water every 3 weeks and 6W receiving 80 mm every 6 weeks. In total, precipitation inputs during the growing season of October 2011 to the end of May 2012 amounted to 614 mm for both treatments.

### Microclimate

Air temperature (*T*_a_) and RH (relative humidity) were continuously measured using EHT sensors with radiation shields (Decagon Devices Inc., Pullman, WA). Water VPD (vapor pressure deficit) was calculated from the temperature and humidity data according to Goudriaan and van Laar (1994). PPFD (Photosynthetic photon flux density) was continuously measured using a QSO‐S PAR sensor (Decagon Devices Inc.). Volumetric soil water content (*θ*_v_), at a depth of 5 cm, was continuously measured in each of the experimental plots using EC‐5 soil moisture sensors (Decagon Devices Inc.). All above‐mentioned sensors were connected to EM‐50 data loggers (Decagon Devices Inc.), recording half‐hourly means. Precipitation was measured with a RG2 rain gauge (Delta‐T Devices, Burwell, Cambridge, U.K.), and stored as half‐hourly means on a DL2 data logger (Delta‐T Devices).

### Plant measurements

Measured plant traits were photosynthetic gas exchange (*A*), stomatal conductance (*g*_s_), transpiration (*E*), predawn and midday leaf water potentials (Ψ_pre_ and Ψ_mid_), predawn and midday maximum quantum yield of photosystem II (F_v_/F_m_), LWC (leaf water content), leaf carbon and nitrogen content (%C and %N), leaf carbon and nitrogen isotope composition (*δ* ^13^C and *δ* ^15^N), and species cover. *A*,* g*_s_, and *E* were measured at midday with a portable open‐flow gas exchange system (LI‐6400; Li‐Cor Inc., Lincoln, NE) at ambient light. WUE (Water‐use efficiency) was calculated as *A*/*g*_s_. Ψ_pre_ and Ψ_mid_ were obtained using a Scholander‐type pressure chamber (Manofrigido, Lisbon, Portugal). F_v_/F_m_ was measured at predawn and midday using a MINI‐PAM portable chlorophyll fluorometer (Walz, Effeltrich, Germany), fitted with a DLC‐8 dark leaf‐clip holder. LWC was determined by comparing fresh weight and dry weight (48 h at 65°C) of a sample of 3–10 fully expanded leaves. These leaf samples were subsequently ground to a fine powder for analysis of %C and %N, and *δ* ^13^C and *δ* ^15^N. These analyses were performed at the Stable Isotopes and Instrumental Analysis Facility of the Centre for Environmental Biology, University of Lisbon, Portugal. The ^13^C/^12^C and ^15^N/^14^N ratios in the samples were determined by continuous flow isotope mass spectrometry (Preston and Owens 1983), on a Sercon Hydra 20–22 (Sercon Ltd., Cheshire, U.K.) stable isotope ratio mass spectrometer, coupled to a EuroEA (EuroVector, Milan, Italy) elemental analyzer for online sample preparation by Dumas‐combustion. The standards used were Sorghum Flour Standard OAS and Wheat Flour Standard OAS (Elemental Microanalysis Ltd., Okehampton, U.K.) for nitrogen and carbon isotope ratios, respectively; *δ* ^15^N results were referred to air and *δ* ^13^C to PeeDee Belemnite. Precision of the isotope ratio analysis, calculated using values from 6 to 9 replicates of laboratory standard material interspersed among samples in every batch analysis, was ±0.2‰.

Species cover was determined in two 38 × 38 cm quadrats in each experimental plot. Within each quadrat, a total of 64 steel pins, 5 cm apart, were inserted vertically into the vegetation. Percent cover for each plant species is calculated by dividing the number of hits by the total number of points within the quadrat.

The above‐mentioned measurements were taken on five species. The studied species include three forbs: (1) *Rumex acetosella* L., a perennial forb, native to the southwestern Mediterranean region, and commonly found in annual grasslands, sprouting in autumn from a spreading rhizome; (2) *Tolpis barbata* (L.) Gaertn., an endemic annual in the Compositae family; and (3) *Tuberaria guttata* (L.) Fourr., an endemic annual in the Cistaceae family. In addition, we studied the legume *Ornithopus sativus* Brot., a species included in the mixture seeded in October 2010 at the experimental site. Finally, we studied the annual grass *Agrostis pourretii* Willd., the most abundant graminoid at the experimental site.

Measurements of plant parameters were taken in the period of February to June. In February, March, and May, data collection was organized around the simultaneous irrigation in 3W and 6W (February 14, March 27, and May 8), with measurements of *A*,* g*_s_, *E*, Ψ, F_v_/F_m_, and LWC being taken 1 day before and one or 2 days after the respective irrigation events. Leaf samples from pre‐ and postwatering were pooled for analysis of tissue carbon and nitrogen, and isotopic composition. In addition, measurements were taken at the end of May, organized around the irrigation in 3W (May 29). At that time, measurements in 3W were taken before and after irrigation. As 6W was not irrigated at the end of May, only one set of data is available for this treatment at that time. Finally, measurements were taken in June (June 12), to assess vegetation performance at the end of the growing season. Not all plant parameters could be assessed at all times in all the five species (Table 1), mainly due to vegetative phase of respective species (e.g., senescence of *Agrostis* in May and June, and *Ornithopus* in June). In addition, leaf‐level gas exchange on *Agrostis* could not be conducted due to the small leaf size.

**Table 1:** Overview of collected data for the five species in the period of February to June. Species indicated by *Rumex* (R), *Tolpis* (To), *Tuberaria* (Tu), *Agrostis* (A), and *Ornithopus* (O)

	February					March					May					End May					June		
F_v_/F_m_	R	To	Tu	A	O	R	To	Tu	A	O	R	To	Tu	A	O			–				–	
Ψ	R	To	–	A	O	R	To	Tu	A	O	R	To	Tu	A	O	R	To	Tu	–	O	R	To	Tu
Leaf water content	R	To	Tu	A	O	R	To	Tu	A	O	R	To	Tu	A	O	R	To	Tu	A	O	R	To	Tu
*A*,* g*_*s*_, *E*	R	To	–	–	O	R	To	Tu	–	O	R	To	Tu	–	O	R	To	Tu	–	O	R	To	Tu
*δ* ^15^N, *δ* ^13^C, %N, %C	R	To	Tu	A	O	R	To	Tu	A	O	R	To	Tu	A	O			–				–	
Cover	R	To	Tu	A	O	R	To	Tu	A	O	R	To	Tu	A	O			–				–	

### Data analysis

Statistical analysis was performed using SigmaPlot 11.0 (Systat Software Inc., San Jose, CA). To assess the effect of treatment on plant performance prior to irrigation, two separate sets of ANOVA (analyses of variance) were performed for each of the measured plant parameters. First of all, data were analyzed separately for each species, using a two‐way repeated‐measures ANOVA, with treatment and time (month) as main effects. In addition, to assess differences among species, data were analyzed separately for each month, using a two‐way ANOVA, with treatment and species as main effects. If data failed to meet the normality assumptions for ANOVA, data were square‐root‐ or log‐transformed. Fisherʼs LSD (least significant difference) *post hoc* pairwise comparison was applied to determine individual differences between means, only when the main effect gave a significant difference.

PCA (Principal component analysis) was used to assess the contribution of physiological plant parameters that discriminate between the responses of individual species, treatment, and irrigation. The integrated responses of the individual species to irrigation were interpreted following the trajectories through multivariate space with time, as previously applied by Potts et al. (2006). PCA was performed using the R function “prcomp” from the “stats” package in R version 3.0.1 (R Development Core Team 2013). Data were mean centered and scaled to 1 standard deviation. PCA was performed for May data collected prior to irrigation, to assess functional differences among the studied species when subjected to moisture deficit. One PCA included state variables Ψ, F_v_/F_m_, and LWC for comparison of all five species. In addition, another PCA was performed for comparison of the three forbs, including not only the above‐mentioned state variables, but also data of leaf‐level gas exchange. Although not presented, equivalent PCA for March showed similar patterns. Finally, PCA was performed separately for each species, over the course of the growing season, including pre‐ and postwatering data.

## Results

### Microclimate and soil water

**Figure 1:**
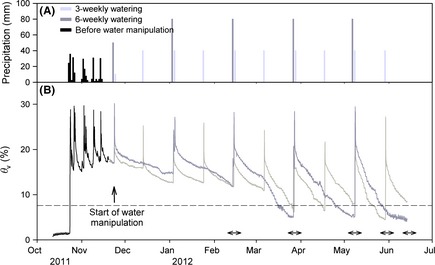
(A) Quantity of water received by the study plots during the growing season of October 2011 to June 2012. All plots received equal water inputs until November 17, when the water manipulation treatment started. 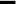 = before water manipulation (natural precipitation), 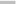 = 3‐weekly watering treatment, 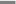 = 6‐weekly watering treatment. (B) Volumetric soil water content (*θ*_v_) at a depth of 5 cm in the study plots for the period of October 2011 to June 2012. 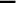 = before water manipulation, 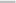 = 3‐weekly watering treatment, 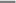 = 6‐weekly watering treatment. Data represent 30‐min values, with *n *=* *4. For clarity, error bars are not shown. Dashed horizontal line represents the soil water content at the permanent wilting point, indicating the threshold for soil water stress. Timing of data collection is indicated by horizontal arrows.

Meteorological conditions at the experimental site over the course of the study are shown in Figure S1. Experimental water manipulation (Fig. 1A) resulted in marked differences in temporal soil moisture dynamics between 3W and 6W (Fig. 1B). From March onwards, supremacy of *θ*_v_ alternated between the two treatments and higher temperatures in combination with increasing biomass and concomitant higher transpiration resulted in a rapid decrease of *θ*_v_ in both treatments. The total number of days during spring with *θ*_v_ below the wilting point was 13 and 42 days in 3W and 6W, respectively. Average *θ*_v_ at 5 cm depth during the February to June period was 11.7 and 11.4% in 3W and 6W, respectively. The variability of soil water content (*θ*_v_), calculated as the coefficient of variation of daily average *θ*_v_ values, increased by 23% (from 0.326 to 0.401) when extending the dry period from 3 to 6 weeks.

### Plant measurements

For a given species, assessed over the course of all measurement periods, treatment differences were apparent (Table S1), for example, Ψ_pre_ in *Tolpis* and *Agrostis*,* E* in *Rumex* and *Tolpis*, and *g*_s_ in *Tuberaria* and *Ornithopus*, elaborated further on below. However, at a given measurement time, statistical analysis did not show differences in treatment effects among species, with a lack of significant interactions between treatment and species in all cases, except for midday F_v_/F_m_ in February (Table S2).

#### Chlorophyll fluorescence

**Figure 2:**
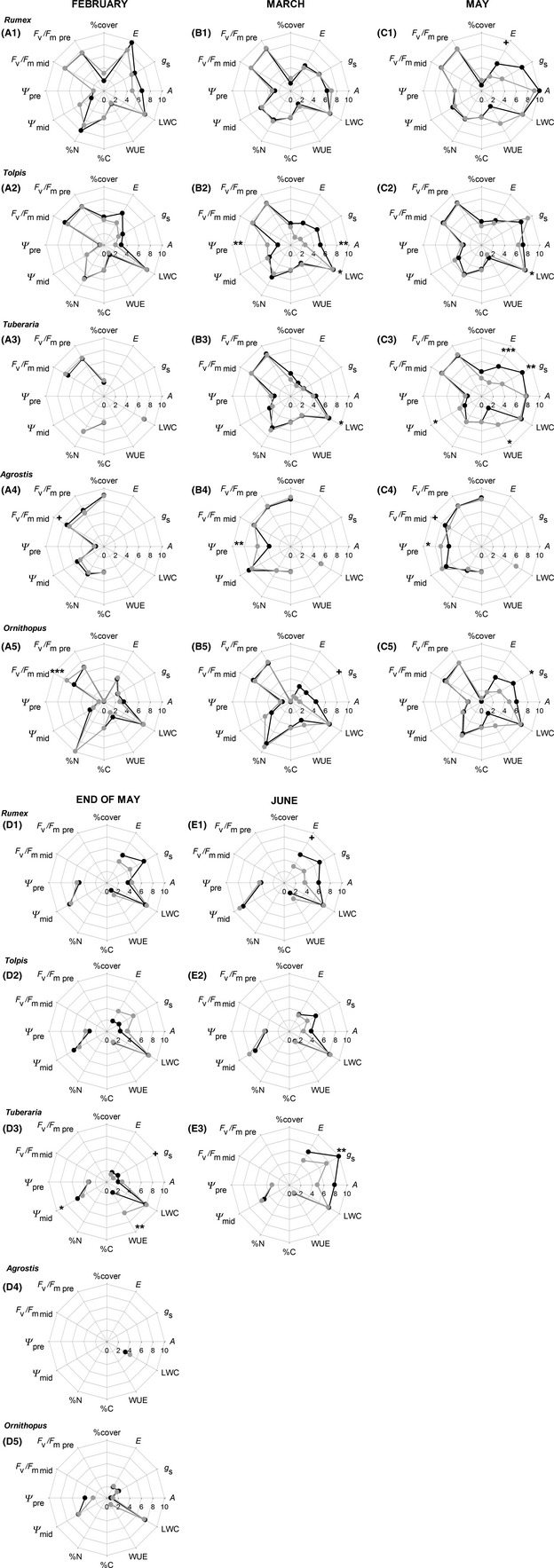
Plant parameters prior to irrigation for the studied species in February (A1–A5), March (B1–B5), May (C1–C5), end of May (D1–D5), and June (E1–E3) in the 3‐weekly (●) and 6‐weekly (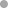) watering treatments. All data were transformed to fall within a 1–10 range. For obtaining actual variable values, following conversion factors need to be used: *A *×* *3 (in *μ*mol m^−2^ sec^−1^), *g*_s_/20 (in mmol m^−2^ sec^−1)^, *E *×* *2 (in mmol m^−2^ sec^−1^), %cover ×10, F_v_/F_m_/10, Ψ/‐4 (in MPa), %N/3, %C × 10, WUE/40, and leaf water content ×10 (in %). *n *=* *4. Asterisks indicate significantly different means (Fisher's least significant difference) between 3‐weekly and 6‐weekly watering treatments (*P <* *0.05, **P <* *0.01, ***P <* *0.001). In addition, 0.05 < *P *<* *0.1 is indicated by **+**.

None of the species showed significant differences in F_v_/F_m_ in response to altered precipitation variability, with the exception of the significant reduction in midday F_v_/F_m_ in *Agrostis* in 6W (Table S1, Fig. 2A4–D4). In all species, values of midday F_v_/F_m_ were typically reduced, as compared to predawn F_v_/F_m_, with the differential of predawn and midday F_v_/F_m_ becoming increasingly larger as the growing season progressed. Both predawn and midday F_v_/F_m_ differed significantly among species, with the forbs generally exhibiting higher F_v_/F_m_ than *Agrostis* and *Ornithopus* (Table S2).

#### Water status

For LWC, a significant, but small treatment difference was found for *Tuberaria* (Table S1, Fig. 2A3–E3). For *Tolpis*, a significant interaction between treatment and month was found (Table S1), with a decrease in LWC in March and May in 6W (Fig. 2B2 and C2). Significant differences among species were observed for LWC over the entire sampling period (Table S2), with *Tolpis* exhibiting the highest (81–86%) and *Agrostis* having the lowest values (42–69%).

In *Tolpis* and *Agrostis*, Ψ_pre_ was significantly lower in 6W, while treatment effects were lacking for Ψ_mid_ in all species (Table S1). Congruent with the seasonal decline in LWC, all species showed significant decreases in both Ψ_pre_ and Ψ_mid_ as the growing season progressed, with the exception of Ψ_pre_ in *Tuberaria*. The grass *Agrostis* exhibited particularly low Ψ_pre_, reaching −1.8 MPa in May (Fig. 2C4).

In the three forbs and in *Ornithopus*, the disparity between Ψ_pre_ and Ψ_mid_ steadily increased during the growing season (Fig. 2), while *Agrostis* exhibited the largest disparity between Ψ_pre_ and Ψ_mid_ in March (up to 1.2 MPa), this difference decreasing to ~0.3 MPa in May, mainly caused by very low Ψ_pre_ with the increase in senescence.

For both Ψ_pre_ and Ψ_mid_, significant differences among species were found (Table S2). *Agrostis* generally exhibited lowest Ψ, while Ψ_pre_ in *Ornithopus* was significantly higher in March and May, as compared to the other species. In June, with *Agrostis* and *Ornithopus* senescent, *Tuberaria* exhibited significantly higher Ψ, as compared to the other two forbs *Rumex* and *Tolpis* (Table S2).

#### Gas exchange

In *Rumex*,* Tuberaria,* and *Ornithopus*,* g*_s_ and *E* were higher in 3W, as compared to 6W, with this difference being significant or nearly significant (Table S1). In contrast, *A* was not significantly altered by treatment in any of these species. For *Tolpis*, no significant treatment effects were found for *g*_s_ and *E*. However, for *A*, a significant interaction between treatment and month was found (Table S1), with a significant decrease of *A* in March in 6W (Fig. 2B2). In all species, *A* reached a maximum in May, with a decline toward the end of May. A recovery in 3W in June, in response to the irrigation at the end of May, was only observed in *Tuberaria* (Fig. 2E3).

Species differences in *A*,* g*_s_, and *E* were significant in all months, with the exception of *g*_s_ and *E* in May (Table S2). Until the end of May, lowest *g*_s_ and *E* were found in *Tuberaria* and *Ornithopus*, while in June *Tuberaria* showed significantly higher values as compared to the other species. On all sampling dates, *Ornithopus* showed lower *A* as compared to the other species, this difference becoming significant at the end of May. In contrast to *Ornithopus,* the three forb species were still active in June, with *Tuberaria* exhibiting significantly higher *A* than *Rumex* and *Tolpis*.

Water‐use efficiency only revealed significant differences in response to precipitation variability in *Tuberaria*, with higher WUE in 6W (Table S1), this increase being apparent in May and at the end of May (Fig. 2C3 and D3). Significant differences in WUE among species were only apparent at the end of May, with the highest WUE in *Tuberaria* while *Ornithopus* exhibited the lowest WUE.

#### Leaf nitrogen (N) and carbon (C) concentration

Neither tissue %N nor %C exhibited significant differences in response to altered precipitation variability in any of the species (Table S1). Differences in tissue %N and %C among species were significant in all months (Table S2), with tissue %N being significantly higher in *Ornithopus*, and significantly lower in *Agrostis*, as compared to the other species. Tissue %C was lowest in *Tolpis*, while *Rumex* had higher tissue %C as compared to the other species (Fig. 2).

#### Cover

Percent cover was not affected by increased precipitation variability in any of the species (Table S1). However, over the course of the growing season, independent of treatment, cover of *Rumex* and *Agrostis* decreased significantly, whereas cover of *Tolpis* and *Ornithopus* was maintained (Fig. 2). In contrast, *Tuberaria* significantly increased its cover with time. Cover percentages for the studied species at the experimental site (ranging from ~2% for *Ornithopus,* and between ~18% and ~39% for *Rumex*,* Tolpis,* and *Tuberaria*, to ~84% for *Agrostis*) reflect the inherent community composition at the experimental site. Throughout the growing season, cover of *Agrostis* was significantly higher as compared to the other species (Table S2), while cover of *Ornithopus* was not only low, but also significantly lower in February and March, as compared to the other species.

#### Isotopic composition

**Figure 3:**
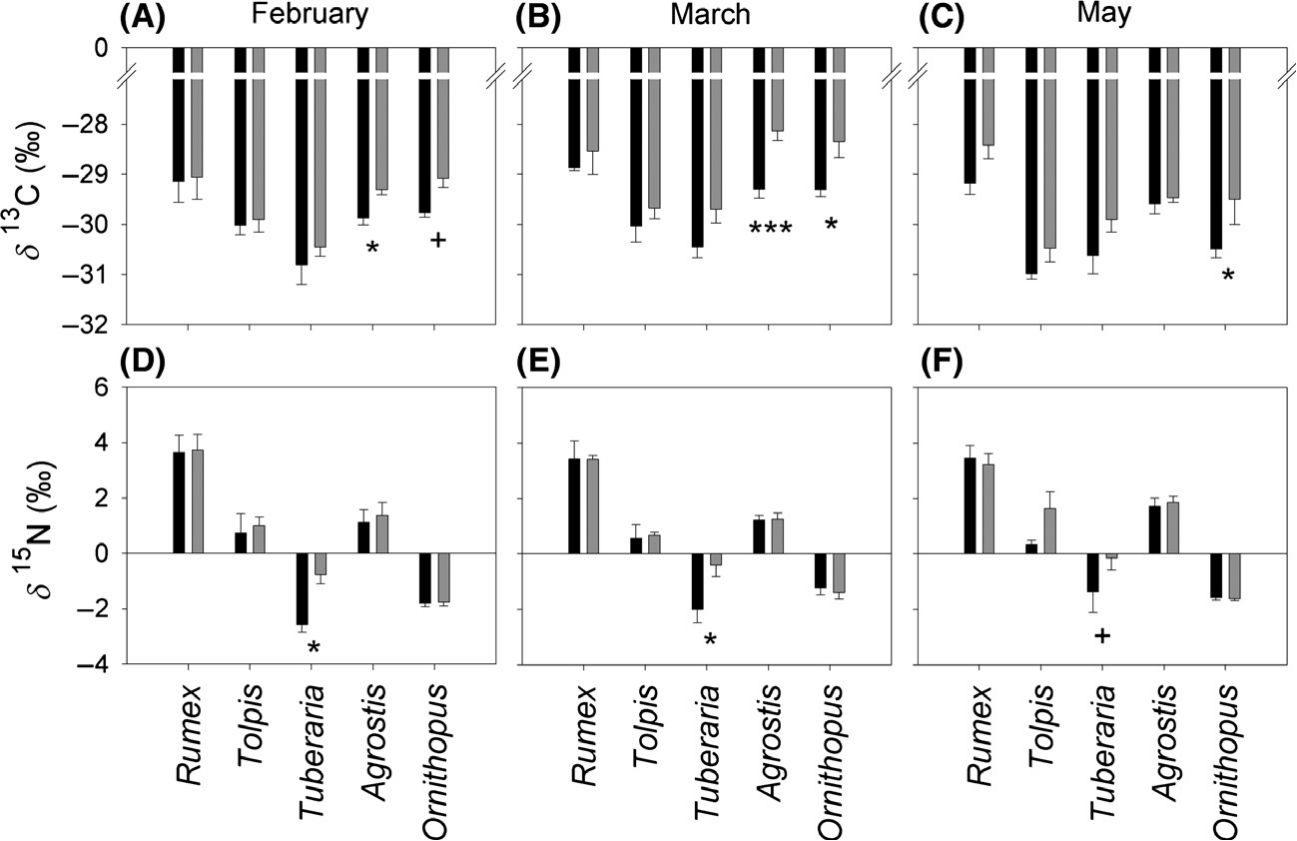
(A–C) *δ* ^13^C and (D–F) *δ* ^15^N in studied species in February, March, and May in the 3‐weekly (■) and 6‐weekly (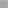) watering treatments. Bars show mean ± SE,* n *=* *4. Asterisks indicate significantly different means (Fisher's least significant difference) between 3‐weekly and 6‐weekly watering treatments (*P <* *0.05, **P <* *0.01, ***P <* *0.001). In addition, 0.05 < *P *<* *0.1 is indicated by +.

Figure 3 shows leaf isotopic compositions (*δ* ^13^C and *δ* ^15^N) of the studied species in February, March, and May. *δ* ^13^C values (Fig. 3A–C) ranged between −28.1 and −31.0‰. In *Rumex*, no significant difference in *δ* ^13^C was found between precipitation treatments, while *Tolpis* and *Tuberaria* exhibited marginally lower *δ* ^13^C in 3W as compared to 6W (*P *=* *0.073 and *P *=* *0.051, respectively, Table S1). In contrast, *δ* ^13^C in *Agrostis* and in *Ornithopus* was significantly depleted in 3W. At all times, a significant difference among species was found for *δ* ^13^C (Table S2). In February, *δ* ^13^C in *Tuberaria* was significantly lower as compared to all other species, while in March both *Tuberaria* and *Tolpis* had significantly stronger ^13^C depletion. In May, *Rumex* showed significantly enriched values in comparison with the other species.

Foliage *δ* ^15^N (Fig. 3D–F) varied between −2.6 and 3.7‰ and was significantly depleted in *Tuberaria* in 3W, as compared to 6W. There was a large and significant species effect on N isotopic composition, with *Rumex* consistently having significantly higher *δ* ^15^N, while *δ* ^15^N in both *Ornithopus* and *Tuberaria* was significantly lower throughout the growing season.

### Principal component analysis

#### PCA based on sampling time

**Figure 4:**
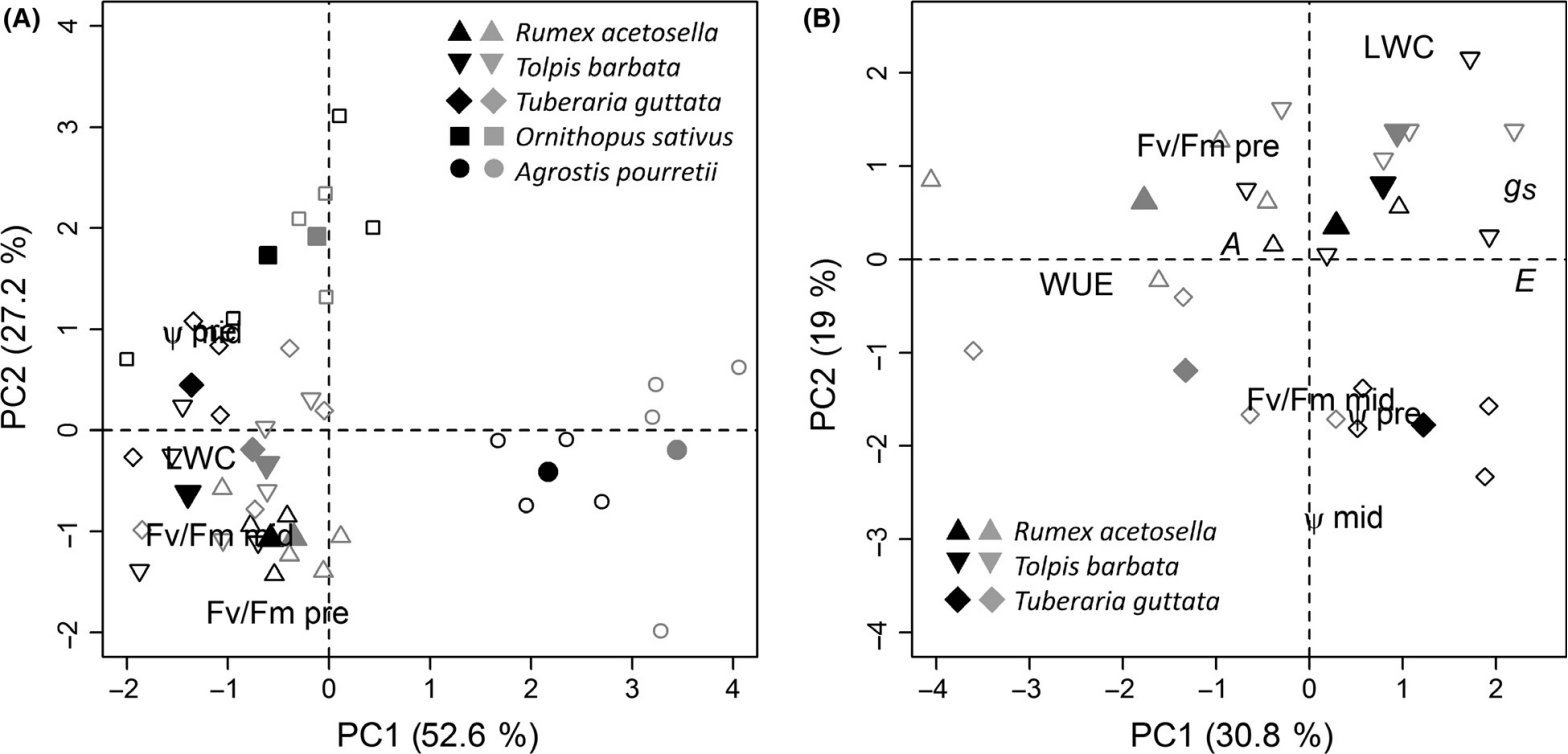
Scatterplots of principal component analyses of plant parameters prior to irrigation in May, showing the first and second PC (principal component) with (A) comparison of all five studied species, and (B) comparison of the three forbs. Symbols: *Rumex acetosella* (▵), *Tolpis barbata* (▽), *Tuberaria guttata* (♢), *Ornithopus sativus* (□), and *Agrostis pourretii* (○) in the 3‐weekly watering treatment (black symbols), and 6‐weekly watering treatment (gray symbols). The individual values are given by open symbols, with the treatment mean indicated by closed symbols. Scaled loading values are indicated by variable names. Percentages of variance explained by the respective PC are given in parentheses in the axes labels.

Functional differences in species prior to irrigation in May were assessed using Ψ, F_v_/F_m_, and LWC as state variables in PCA (Fig. 4A). *Agrostis* is clearly separated from the other species on PC1, exhibiting low parameter values. *Rumex*,* Tolpis*, and *Tuberaria* are clustered together, while separation is evident between these forbs and *Ornithopus* on PC2, the latter species having higher *ψ*. Within species, the separation between treatments is almost consistently represented on PC1. However, treatment differences were small, with the exception of *Agrostis*.

Including all state variables, functional differences among the forbs are evident (Fig. 4B), with high values for predawn F_v_/F_m_ and *A* in *Rumex*, high Ψ in *Tuberaria*, and high LWC and *g*_s_ in *Tolpis*. Treatment differences in *Rumex* and *Tuberaria* were apparent on PC1; that is, in 6W these species were characterized by high WUE, and low *g*_s_ and *E*. However, for *Tolpis*, treatment differences were small.

#### PCA based on individual species

**Figure 5:**
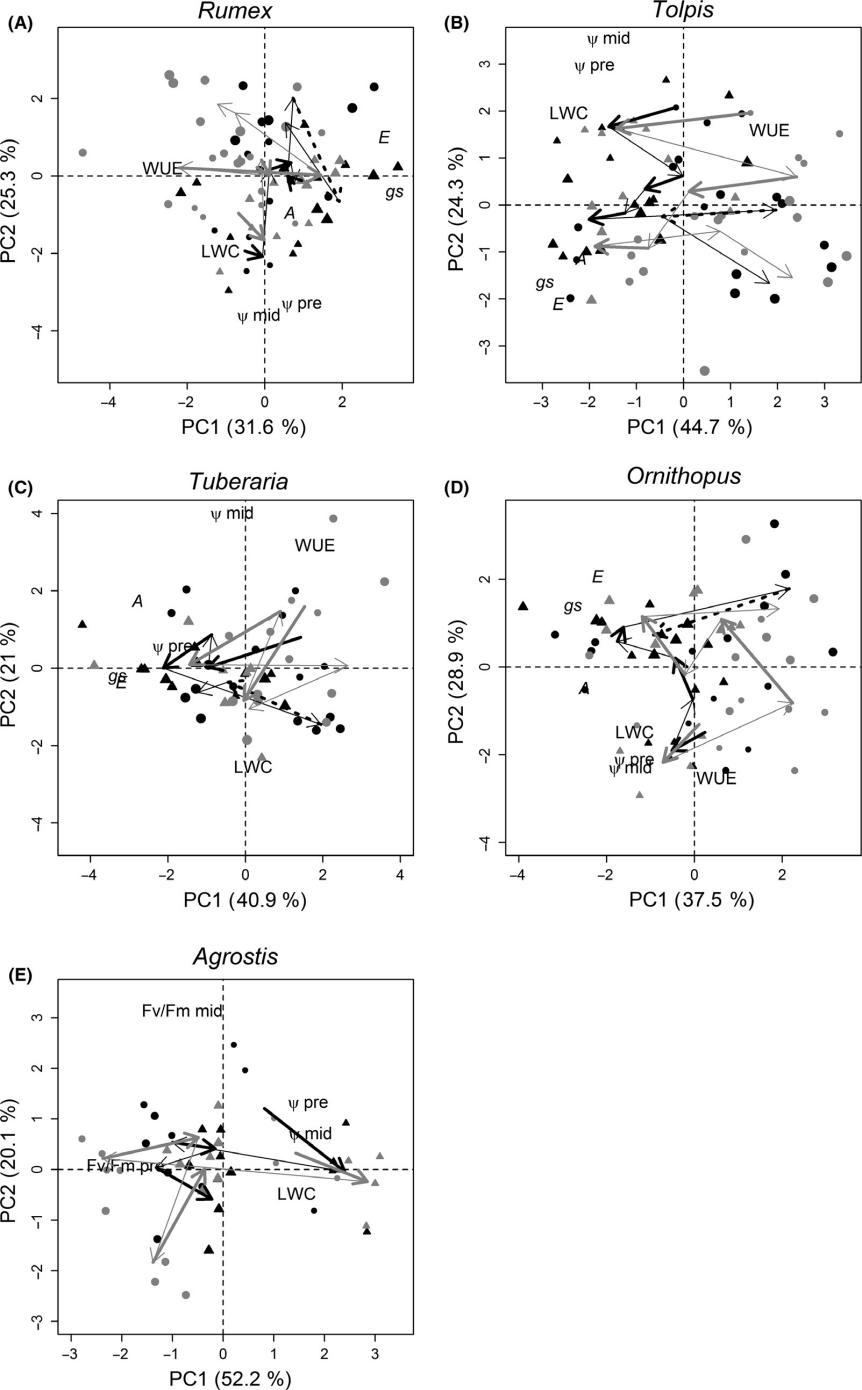
Scatterplots of principal component analyses of plant parameters over the course of the growing season, showing the first and second PC (principal component) for (A) *Rumex acetosella*, (B) *Tolpis barbata*, (C) *Tuberaria guttata*, (D) *Ornithopus sativus*, and (E) *Agrostis pourretii*. Symbols: ● and 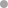 represent before irrigation in the 3‐ and 6‐weekly watering treatments, respectively; ▲ and 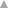 represent after irrigation in the 3‐ and 6‐weekly watering treatment, respectively. All individual values are given, with the arrowed lines connecting means of consecutive measurements, with symbol size increasing with time in year. Thick lines represent the trajectory shifts with irrigation, and thin lines represent the trajectory shifts from after irrigation to the consecutive before irrigation state. Thick dashed lines represent the shifts with irrigation in the 3‐weekly watering treatment at the end of May. Scaled loading values of state variables are indicated. Percentages of variance explained by the respective PC are given in parentheses in the axes labels. State variables used and trajectories included depended on available complete data sets for the respective species (Table 1). Trajectories start in February, with the exception of *Tuberaria*, which starts in March (due to the absence of data on Ψ in February). Trajectories of *Agrostis* stop in May, with no data available for the end of May and June.

For the forb species and *Ornithopus*, PC1 was associated with changes in *g*_s_ and either *E* or *A*, thus representing parameters related to photosynthesis. PC2 was associated with leaf water status, correlating with changes in Ψ and LWC, and also with WUE in *Tuberaria* and *Ornithopus* (Fig. 5A–D).

For all of these four species, ordinations from 3W and 6W often did cluster, especially after irrigation, indicating only small treatment differences. However, treatment differences could be observed along PC1 prior to irrigation in May for *Rumex* and *Tuberaria*, in March for *Tolpis*, and in March and May for *Ornithopus*, showing lower values for photosynthesis‐related parameters in 6W, as compared to 3W. Irrigation in *Tolpis*,* Tuberaria*, and *Ornithopus* resulted in large shifts in multivariate space, increasing *E* and *g*_s_ as well as, for *Tolpis*,* A*, and decreasing WUE. For *Rumex*,* Ornithopus*, and most clearly for *Tolpis*, the multivariate trajectory over the course of the growing season moves along PC2, toward lower values of Ψ. However, for *Tuberaria*, seasonality was not clearly reflected in PCA.

For *Agrostis*, where a different set of predictors was used, PC1 spans between predawn F_v_/F_m_ and Ψ, and PC2 correlates positively with midday F_v_/F_m_ (Fig. 5E). Samples from February appeared to be distinct from the other months, forming a cluster with higher values on PC1. Prior to irrigation, treatments were separated. However, after irrigation, ordinations did cluster, indicating only small treatment differences. The effect of irrigation can be seen as a movement along PC1, indicating a recovery of leaf water status. For *Agrostis*, no clear trajectory can be observed along the growing season.

## Discussion

### Species‐specific adaptation strategies – the grass *Agrostis pourretii*

In *Agrostis*, the consistently lower midday F_v_/F_m_ in 6W indicates a higher dynamic photo‐inhibition with increasing precipitation variability, which suggests decreasing efficiency of photochemical and nonphotochemical quenching (Björkman and Demmig 1987; Johnson et al. 1993). In addition, *Agrostis* exhibited significantly lower Ψ_pre_ in March and May in 6W, indicating a decline of nocturnal water status recovery, probably due to its shallow root system. However, no differences were found for Ψ_mid_, suggesting the ability of tight short‐term stomatal control in response to low *θ*_v_. This is supported by higher *δ* ^13^C in 6W, indicating higher WUE (Farquhar et al. 1989). The observed differences in physiological characteristics of *Agrostis* with increasing precipitation variability are indicative for isohydric plants (Stocker 1956), with tight short‐term stomatal control and a minimum threshold of Ψ, thereby managing water loss through stomata, accompanied by higher WUE. However, irrigation resulted in an opportunistic response in *Agrostis*, with the trajectories in multivariate space driven by a rapid increase in leaf water status, decreasing the divergence between the two treatments. This behavior is indicative of a water spender strategy with high growth rates and a rapid life cycle closely coupled to water availability. This strategy has previously been reported for annual grass species in Mediterranean environments, showing drought resistance, in combination with a water spender mechanism upon irrigation (Bicak and Sternberg 1993; Ramírez et al. 2008; Chirino et al. 2011). In our study, *Agrostis* was the most abundant species, which might reflect the general success of its physiological adaptations to fluctuations in water availability.

Our data do not suggest adaptive structural changes in *Agrostis* as precipitation variability increases, with no treatment effects on tissue C and N. As the photosynthetic apparatus is the largest sink for nitrogen in foliage (Evans and Seemann 1989), the low tissue N in *Agrostis* is probably due to a dilution effect of growth (Walker et al. 2001), resulting from a combination of low soil‐N content (Jongen et al. 2013b) and high growth rates. In addition, independent of treatment, this species exhibited a rapid curtailment of vegetative growth in May, allocating resources to reproductive organs, and accelerating leaf senescence, the latter also evident from the rapidly decreasing LWC in May in both treatments.

### Species‐specific adaptation strategies– the forbs

With none of the studied forb species exhibiting treatment‐related photo‐inhibition, different adaptation strategies were observed among the three forb species, extrapolated from data on leaf‐level gas exchange and leaf water status. In *Rumex*, changing precipitation patterns did not result in significant treatment differences in F_v_/F_m_, Ψ, *A*,* g*_s_, and WUE prior to irrigation, the latter confirmed by the lack of a treatment effect on *δ* ^13^C. Thus, the adaptation to moisture deficit in *Rumex* may be found in its innate morphological characteristics, that is, leaf succulence, with maintenance of leaf water status and stomatal aperture, thereby preventing CO_2_ limitation of photosynthesis. Following the trajectories through multivariate space with irrigation confirms the good adaptation of *Rumex* to variability in soil moisture, with irrigation only resulting in small shifts in both treatments. To date, relatively few studies on drought tolerance in *Rumex acetosella* exist (i.e., Zimmerman and Lechowicz 1982; Farris 1984; Houssard et al. 1992; Mamolos et al. 2001). Houssard et al. (1992) showed that *Rumex* responds to water deficit by restricting water loss, suggesting a physiological basis of drought tolerance. Indeed, in May, *Rumex* exerted some degree of stomatal control, with a decrease in *g*_s_ and concomitantly higher WUE in 6W, reinforcing the capacity for isohydric behavior. *Tuberaria* showed typical isohydric behavior with treatment differences prior to irrigation revealing a good stomatal control in response to low *θ*_v_, maintaining a constant Ψ and higher WUE in 6W, the latter also reflected in higher *δ* ^13^C (*P *=* *0.051). This was accompanied by a decrease in LWC, suggesting that *g*_s_ was more closely linked to low *θ*_v_ than to leaf water status. The trajectories through multivariate space with irrigation were dictated by changes in *E* and *g*_s_. In addition, the response to irrigation in 6W was characterized by increasing LWC. The finding that *δ* ^15^N signatures in *Tuberaria* were similar to the legume *Ornithopus*, while being markedly depleted as compared to the other species, may indicate facilitative legume neighbor interactions, with the treatment effect on *δ* ^15^N in *Tuberaria* caused by a decrease in facilitation with moisture deficit, as reported previously by Khan et al. (2014).

*Tolpis* showed little evidence of physiological adaptations supporting isohydric behavior in response to soil moisture deficits, with *A* corresponding directly to altered *g*_s_, and consequently low WUE. However, morphological adaptations, that is, the taproot system, distinctive for this species, can delay the onset of water stress, thereby maintaining leaf water status (Persson and Baitulin 1996). Nevertheless, this strategy was not evident in March, with the decrease in Ψ_pre_ in 6W indicating a decline of nocturnal water recharge, probably due to the late development of an effective taproot. The trajectories with irrigation in *Tolpis* were, especially in 6W, dictated by a rapid increase in both *A* and *g*_s._ Indeed, studies have shown that recovery of photosynthetic capacity upon irrigation can be rapid, with attainment of predrought photosynthetic rates (Xu et al. 2009; Zlatev 2013). In *Tolpis*, the rapid recovery indicates that the depression of *A* with moisture deficit can be attributed to stomatal limitation of CO_2_ (Cornic 2000), as studies have shown that metabolic limitations to photosynthesis are reversed considerably slower (Ignace et al. 2007; Galle et al. 2009; Hu et al. 2010).

Independent of treatment, differences in plant parameters were observed among the three forb species. With the exception of June, *Rumex* attained highest *A*, although this was not translated into an increase in cover. In this species, high carbon investments into rhizome biomass can be expected (Mooney 1972; Xiong and Katterer 2010). In addition, the structural carbon content in the succulent leaves was higher as compared to the other species. In June, *Tuberaria* attained highest A, with maintenance of leaf water status, reflecting the supremacy of its typical isohydric behavior.

### Species‐specific adaptation strategies – the legume *Ornithopus sativus*

Extensive literature is available on productivity of *Ornithopus sativus* (e.g., de Lautour and Rumball 1986; Iglesias and Lloveras 2000; Nichols et al. 2007), a species often included in legume seed mixtures for pasture improvement (Nichols et al. 2007). Performance of this species with moisture deficit has received little attention to date, bar the mentioning of the absence of symptoms to water stress (Wickham et al. 2007). In relation to increasing precipitation variability, previous comparison among functional group abundance revealed a higher sensitivity of legumes to altered precipitation regimes (Jongen et al. 2013b).

Ecophysiological variables for *Ornithopus* did show an increased susceptibility to low *θ*_v_, although significant treatment effects were only found for *g*_s_ and *δ* ^13^C, with decreasing *g*_s_ in March and May, and higher *δ* ^13^C at all times in 6W. However, with soil moisture deficit in 6W in March and May, the decrease in *g*_s_ was accompanied by a decrease in *A*, this difference being significant in March (t‐test: *P *=* *0.004). Moreover, the intolerance of *Ornithopus* to low *θ*_v_ was evident from the large treatment separation in multivariate space in March and May prior to irrigation. However, irrigation resulted in a larger shift in 6W, as compared to 3W, indicating rapid recovery of leaf water status and photosynthetic apparatus. In comparison with the forbs, *Ornithopus* showed lower *A* and displayed a rapid phenological development, initiating senescence in May. In addition, *g*_s_ and tissue C were comparable to *Tuberaria*, which is in line with maintenance of high Ψ throughout the season. However, in contrast to *Tuberaria*, this did not result in increased photosynthesis and growth, likely due to the effects of increased photo‐inhibition, as indicated by substantially lower midday F_v_/F_m_.

In *Ornithopus,* the rhizo‐symbiotic nitrogen acquisition pattern, typical for legumes, was evident from the significantly lower tissue *δ* ^15^N, as compared to *Rumex*,* Tolpis,* and *Agrostis*, and the significantly higher tissue N, as compared to all other species. However, although studies have shown that N_2_ fixation in legumes is sensitive to moisture deficiency (e.g., Serraj et al. 1999; Vicente et al. 2012), the lack of treatment effects on either tissue N or *δ* ^15^N does not suggest an increase in water stress on *Rhizobium* with increasing precipitation variability.

### Vegetation resilience to changing precipitation variability

Increasing precipitation variability, extending the dry period between precipitation events from 3 to 6 weeks, did lead to increased soil moisture deficits. However, aboveground productivity in the understorey of this Mediterranean oak woodland was resilient to increased precipitation variability (Jongen et al. 2013b). Our results confirmed this resilience, with sustainment of photosynthetic performance with temporarily decreased *θ*_v_ in *Rumex*,* Tolpis*,* Tuberaria,* and *Agrostis*, these species accounting for 61% of aboveground productivity and representing the functional groups of forbs and grasses. Indeed, these two functional groups have previously been shown to be unaffected by increased precipitation variability (Jongen et al. 2013b). The present study confirms this finding and explains it with adaptive physiological mechanisms, that is, isohydric behavior in *Agrostis*,* Rumex,* and *Tuberaria*, and suggests probable morphological adaptations, that is, succulence in *Rumex* and taproots in *Tolpis*. These adaptation mechanisms thus sustained the performance of these species when water was scarce. In addition, quick recovery upon irrigation events and species‐specific adaptations of water‐use efficiency with longer dry periods and larger precipitation events contributed to the observed resilience in productivity.

The legume *Ornithopus* was sensitive to moisture deficit, with no distinct adaptation mechanism detectable. This is in line with Jongen et al. (2013b), reporting lower productivity for the functional group of legumes with increasing precipitation variability. However, with legumes only accounting for a small percentage of total productivity, this did not impair ecosystem resilience to changing precipitation patterns. Nevertheless, this may have implications for the agricultural practice of seeding legume‐rich mixtures, and justifies additional research for drought‐tolerant cultivar improvement (i.e., Erice et al. 2010; Real et al. 2011) to maintain productivity with climate change.

Although precipitation manipulation treatments have been applied during the November to May period for three consecutive years on the same experimental plots (weekly vs. 3‐weekly watering treatments, starting in 2009 and 2010, and 3‐weekly vs. 6‐weekly watering treatments, starting in 2011), no significant effects of increased precipitation variability on cover of the studied species were found in the present study. This may suggest no changes in community structure, with adaptational success in response to temporarily low soil moisture not altering competitive interactions among the coexisting plant species. Shifts in species composition and diversity with environmental change have previously been related to differences in productivity of the dominant species present (e.g., Klanderud and Totland 2005; Kardol et al. 2010; Báez et al. 2013). For example, plant community responses to altered precipitation quantity were linked to morphological traits of the dominant species (Sternberg et al. 1999). Further, in relation to increasing precipitation variability, Knapp et al. (2002) reported increasing diversity in a mesic grassland, with a concomitant productivity decrease in the dominant grass species. The lack of treatment effects on cover in our study may be partially attributed to the relatively short period of manipulation. Shifts in species composition may only occur in the long term and are thus rarely observed in short‐term precipitation manipulation studies (Weltzin et al. 2003). Indeed, in a recent review it was shown that the majority of studies did not detect changes in community structure and biodiversity with decreasing precipitation frequency, which was explained by the short duration of most studies and by the degree of manipulation lying within the range of natural precipitation variability encountered (Unger and Jongen 2015). However, even in a long‐term experiment (9 years), with manipulation of the amount of precipitation, Tielbörger et al. (2014) did not find treatment differences in species composition, richness, and density in a Mediterranean and semi‐arid ecosystem. They argue that this resistance is most likely explained by the fact that the component species have developed a variety of adaptation mechanisms to the naturally large variation in precipitation (Tielbörger et al. 2014), as indeed, is suggested from results in the present study.

Nevertheless, long‐term effects of altered precipitation regimes on, for example, shifts in competition intensity or seed bank regeneration, consequently affecting ecosystem productivity and community structure in subsequent years, cannot be excluded. This emphasizes the need for long‐term precipitation manipulation experiments to capture possible directional changes of species composition, which can subsequently affect ecosystem state and functioning. Answers to these questions are needed to develop appropriate land management practices and mitigation strategies in the face of increasing climatic change and variability.

## Conflict of Interest

None declared.

## Supporting information


**Figure S1**. Daily average air temperature (˚C, ∙∙∙∙), average daytime (11.00‒18.00 h) vapor pressure deficit (VPD in kPa, ─) and daily‐integrated photosynthetic photon flux density (PPFD in mol m^−2^ d^−1^, █) at the experimental site over the course of the study.
**Table S1**. Summary of two‐way repeated measures ANOVA, with factors treatment (T) and month (M) of all plant parameters prior to irrigation in the studied species. For M, results of Fisherʼs LSD are indicated, with different letters indicating significantly different means (*P *<* *0.05) for February, March, May, end of May, and June.
**Table S2**. Summary of two‐way ANOVA, with factors treatment (T) and species (S) of all plant parameters prior to irrigation in February, March, May, end of May and June. For S, results of Fisherʼs LSD are indicated, with different letters indicating significantly different means (*P *<* *0.05) for *Rumex*,* Tolpis*,* Tuberaria*,* Agrostis* and *Ornithopus*.
