# Supplementary material for: Species‐specific adaptations explain resilience of herbaceous understorey to increased precipitation variability in a Mediterranean oak woodland
Source: Ecol Evol. 2015 Sep 9;5(19):4246–62. doi: 10.1002/ece3.1662 (PMC4667836; doi:10.1002/ece3.1662)
Supplement: Supplementary file 1 — Figure S1. Daily average air temperature (˚C, ∙∙∙∙), average daytime (11.00‒18.00 h) vapor pressure deficit (VPD in kPa, ─) and daily‐integrated photosynthetic photon flux density (PPFD in mol m−2 d−1, █) at the experimental site over the course of the study. Table S1. Summary of two‐way repeated measures ANOVA, with factors treatment (T) and month (M) of all plant parameters prior to irrigation in the studied species. For M, results of Fisherʼs LSD are indicated, with different letters indicating significantly different means (P < 0.05) for February, March, May, end of May, and June. Table S2. Summary of two‐way ANOVA, with factors treatment (T) and species (S) of all plant parameters prior to irrigation in February, March, May, end of May and June. For S, results of Fisherʼs LSD are indicated, with different letters indicating significantly different means (P < 0.05) for Rumex, Tolpis, Tuberaria, Agrostis and Ornithopus. [file ECE3-5-4246-s001.docx]

**SUPPLEMENTARY MATERIAL**

**Species-specific adaptations explain resilience of herbaceous understorey to increased precipitation variability in a Mediterranean oak woodland**

Marjan Jongen, Christine Hellmann, Stephan Unger,





Figure S1 - Daily average air temperature (˚C, ∙∙∙∙), average daytime (11.00‒18.00h) vapour pressure deficit (VPD in kPa, ─) and daily-integrated photosynthetic photon flux density (PPFD in mol m^-2^ d^-1^, █) at the experimental site over the course of the study.

Daily average air temperature at the experimental site over the course of the study ranged from 3.9°C to 25.9°C, with daily-integrated PPFD varying between 3.1 and 62.1 mol m^-2^ d^-1^. March was relatively warm, and characterized by two warm spells in the period of March 9-14 and March 25-28, with above-average daily maximum temperatures ranging from 24.4 to 27.9°C, and VPD between 1.8 and 2.5 kPa. May saw a rapid increase in temperature, with daily maximum temperature in the period of May 8 to June 13 exceeding 30°C on 15 occasions, with concomitant high VPD (1.6-3.6 kPa).

Table S1 - Summary of two-way repeated measures ANOVA, with factors treatment (T) and month (M) of all plant parameters prior to irrigation in the studied species. For M, results of Fisherʼs LSD are indicated, with different letters indicating significantly different means (*p* < 0.05) for February, March, May, end of May, and June.

*Rumex acetosella*

|  | F_v_/F_m pre_ | F_v_/F_m mid_ | Ψ_pre_ | Ψ_mid_ | LWC | *A* | *g*_s_ | *E* | WUE | δ^15^N | δ^13^C | %N | %C | %cover |
| --- | --- | --- | --- | --- | --- | --- | --- | --- | --- | --- | --- | --- | --- | --- |
| T | F_1,12_=0.65 *p*=0.45 | F_1,12_=0.01 *p*=0.91 | F_1,24_=1.70 *p*=0.24 | F_1,23_=2.99 *p*=0.13 | F_1,22_=0.51 *p*=0.50 | F_1,21_=0.72  *p*=0.43 | F_1,21_=4.69  *p*=0.072 | F_1,21_=7.23  *p*=0.034 | F_1,21_=2.66  *p*=0.15 | F_1,11_=0.01  *p*=0.91 | F_1,11_=0.91  *p*=0.38 | F_1,11_=0.05  *p*=0.83 | F_1,11_=0.08  *p*=0.78 | F_1,12_=0.55  *p*=0.49 |
| M | F_2,12_=108 *p*<0.001  a,b,b,-,- | F_2,12_=0.65 *p*=0.54 | F_4,24_=19.3 *p*<0.001  a,b,c,c,c | F_4,23_=13.5 *p*<0.001  a,bc,b,cd,d | F_4,22_=6.00 *p*=0.002  a,b,a,b,b | F_4,21_=14.2  *p*<0.001  ab,a,c,d,bd | F_4,21_=0.05  *p*=0.99 | F_4,21_=3.12  *p*=0.034  a,b,ab,b,b | F_4,21_=3.63  *p*=0.021  a,ab,b,a,a | F_2,11_=2.71  *p*=0.11 | F_2,11_=1.25  *p*=0.33 | F_2,11_=29.3  *p<*0.001  a,b,b,-,- | F_2,11_=4.55  *p*=0.036  a,ab,b,-,- | F_2,12_=5.19  *p*=0.024  a,ab,b,-,- |
| T×M | F_2,12_=0.40  *p*=0.68 | F_2,12_=0.11  *p*=0.90 | F_4,24_=0.15  *p*=0.96 | F_4,23_=1.24  *p*=0.32 | F_4,22_=2.25  *p*=0.097 | F_4,21_=1.55  *p*=0.23 | F_4,21_=0.66  *p*=0.63 | F_4,21_=0.59  *p*=0.67 | F_4,21_=0.74  *p*=0.58 | F_2,11_=0.24  *p*=0.79 | F_2,11_=0.63  *p*=0.55 | F_2,11_=4.01  *p*=0.049 | F_2,11_=0.97  *p*=0.41 | F_2,12_=0.36  *p*=0.71 |

*Tolpis barbata*

|  | F_v_/F_m pre_ | F_v_/F_m mid_ | Ψ_pre_ | Ψ_mid_ | LWC | *A* | *g*_s_ | *E* | WUE | δ^15^N | δ^13^C | %N | %C | %cover |
| --- | --- | --- | --- | --- | --- | --- | --- | --- | --- | --- | --- | --- | --- | --- |
| T | F_1,12_=1.79 *p*=0.23 | F_1,12_=2.23 *p*=0.19 | F_1,24_=8.22 *p*=0.029 | F_1,24_=1.31 *p*=0.30 | F_1,21_=1.81 *p*=0.22 | F_1,21_=3.30 *p*=0.12 | F_1,21_=0.12 *p*=0.74 | F_1,21_=1.24 *p*=0.30 | F_1,21_=0.00 *p*=0.98 | F_1,11_=1.97  *p*=0.21 | F_1,12_=4.71  *p*=0.073 | F_1,12_=0.19  *p*=0.68 | F_1,12_=0.12  *p*=0.75 | F_1,12_=0.19  *p*=0.68 |
| M | F_2,12_=29.1  *p*<0.001  a,b,b,-,- | F_2,12_=0.09 *p*=0.91 | F_4,24_=27.2 *p*<0.001  a,b,b,b,c | F_4,24_=20.4 *p*<0.001  a,b,b,c,d | F_4,21_=18.5 *p*<0.001  ab,a,b,a,c | F_4,21_=18.9 *p*<0.001  a,b,c,ab,ab | F_4,21_=4.02 *p*=0.014  a,a,b,a,a | F_4,21_=4.14 *p*=0.013  a,ab,c,bc,bc | F_4,21_=4.09 *p*=0.013  a,b,a,a,a | F_2,11_=0.21  *p*=0.82 | F_2,12_=6.86  *p*=0.010  a,a,b,-,- | F_2,12_=1.34  *p=*0.30 | F_2,12_=1.36  *p*=0.29 | F_2,12_=3.73  *p*=0.055 |
| T×M | F_2,12_=0.05  *p*=0.95 | F_2,12_=2.18  *p*=0.16 | F_4,24_=2.09  *p*=0.11 | F_4,24_=1.67  *p*=0.19 | F_4,21_=3.39  *p*=0.027 | F_4,21_=3.26  *p*=0.032 | F_4,21_=1.36  *p*=0.28 | F_4,21_=2.57  *p*=0.068 | F_4,21_=0.28  *p*=0.89 | F_2,11_=2.06  *p*=0.17 | F_2,12_=0.30  *p*=0.75 | F_2,12_=0.46  *p*=0.64 | F_2,12_=1.32  *p*=0.30 | F_2,12_=0.04  *p*=0.96 |

*Tuberaria guttata*

|  | F_v_/F_m pre_ | F_v_/F_m mid_ | Ψ_pre_ | Ψ_mid_ | LWC | *A* | *g*_s_ | *E* | WUE | δ^15^N | δ^13^C | %N | %C | %cover |
| --- | --- | --- | --- | --- | --- | --- | --- | --- | --- | --- | --- | --- | --- | --- |
| T | F_1,11_=0.00 *p*=0.97 | F_1,12_=2.28 *p*=0.18 | F_1,18_=1.49 *p*=0.28 | F_1,18_=0.02 *p*=0.89 | F_1,24_=6.79 *p*=0.040 | F_1,15_=2.55 *p*=0.14 | F_1,15_=13.5  *p*=0.007 | F_1,15_=7.82 *p*=0.028 | F_1,15_=7.40 *p*=0.029 | F_1,12_=10.3  *p*=0.018 | F_1,12_=5.91  *p*=0.051 | F_1,12_=0.06  *p*=0.81 | F_1,12_=0.00  *p*=0.95 | F_1,12_=0.07  *p*=0.80 |
| M | F_2,11_=15.8  *p*<0.001  a,b,b,-,- | F_2,12_=2.84 *p*=0.098 | F_3,18_=0.66 *p*=0.59 | F_3,18_=16.2 *p*<0.001  -,a,a,b,b | F_4,24_=5.37 *p*=0.003  a,b,a,a,a | F_3,15_=12.3 *p*<0.001  -,a,b,a,b | F_3,15_=39.3 *p*<0.001  -,a,b,a,c | F_3,15_=25.3 *p*<0.001  -,a,b,a,c | F_3,15_=3.23 *p*=0.053 | F_2,12_=2.46  *p*=0.13 | F_2,12_=2.16  *p*=0.16 | F_2,12_=24.9  *p<*0.001  a,a,b,-,- | F_2,12_=4.86  *p*=0.028  a,a,b,-,- | F_2,12_=11.6  *p*=0.002  a,b,b,-,- |
| T×M | F_2,11_=1.65  *p*=0.24 | F_2,12_=1.29  *p*=0.31 | F_3,18_=0.20  *p*=0.89 | F_3,18_=5.17  *p*=0.009 | F_4,24_=1.60  *p*=0.21 | F_3,15_=1.55  *p*=0.24 | F_3,15_=1.08  *p*=0.39 | F_3,15_=2.40  *p*=0.11 | F_3,15_=1.99  *p*=0.16 | F_2,12_=0.27  *p*=0.77 | F_2,12_=0.32  *p*=0.73 | F_2,12_=0.55  *p*=0.59 | F_2,12_=1.94  *p*=0.19 | F_2,12_=5.81  *p*=0.017 |

*Agrostis pourretii*

|  | F_v_/F_m pre_ | F_v_/F_m mid_ | Ψ_pre_ | Ψ_mid_ | LWC | δ^15^N | δ ^13^C | %N | %C | %cover |
| --- | --- | --- | --- | --- | --- | --- | --- | --- | --- | --- |
| T | F_1,12_=5.18 *p*=0.063 | F_1,9_=7.64 *p*=0.030 | F_1,12_=24.0 *p*=0.003 | F_1,12_=0.10 *p*=0.76 | F_1,18_=2.11 *p*=0.20 | F_1,10_=0.13  *p*=0.73 | F_1,11_=13.2  *p*=0.011 | F_1,11_=0.68  *p*=0.44 | F_1,11_=4.51  *p*=0.072 | F_1,12_=0.00  *p*=0.98 |
| M | F_2,12_=32.4  *p*<0.001  a,b,b,-,- | F_2,9_=1.22 *p*=0.34 | F_2,12_=67.4 *p*<0.001  a,b,c,-,- | F_2,12_=23.3 *p*<0.001  a,b,b,-,- | F_3,18_=23.0 *p*<0.001  a,b,a,c,- | F_2,10_=2.16  *p*=0.17 | F_2,11_=30.9  *p*<0.001  a,b,a,-,- | F_2,11_=3.72  *p*=0.058 | F_2,11_=0.59  *p*=0.57 | F_2,12_=4.38  *p*=0.037  a,b,b,-,- |
| T×M | F_2,12_=2.83  *p*=0.099 | F_2,9_=0.83  *p*=0.47 | F_2,12_=2.14  *p*=0.16 | F_2,12_=1.51  *p*=0.26 | F_3,18_=1.38  *p*=0.28 | F_2,10_=0.13  *p*=0.88 | F_2,11_=9.00  *p*=0.005 | F_2,11_=0.39  *p*=0.68 | F_2,11_=0.03  *p*=0.97 | F_2,12_=1.45  *p*=0.27 |

*Ornithopus sativus*

|  | F_v_/F_m pre_ | F_v_/F_m mid_ | Ψ_pre_ | Ψ_mid_ | LWC | *A* | *g*_s_ | *E* | WUE | δ^15^N | δ ^13^C | %N | %C | %cover |
| --- | --- | --- | --- | --- | --- | --- | --- | --- | --- | --- | --- | --- | --- | --- |
| T | F_1,11_=0.31 *p*=0.87 | F_1,12_=1.28 *p*=0.30 | F_1,18_=1.49 *p*=0.27 | F_1,18_=0.62 *p*=0.46 | F_1,16_=1.72 *p*=0.24 | F_1,18_=3.87 *p*=0.097 | F_1,18_=6.65 *p*=0.042 | F_1,18_=5.85 *p*=0.052 | F_1,18_=1.05 *p*=0.35 | F_1,12_=0.09  *p*=0.77 | F_1,12_=10.5  *p*=0.018 | F_1,12_=0.34  *p*=0.58 | F_1,12_=1.23  *p*=0.31 | F_1,12_=0.01  *p*=0.92 |
| M | F_2,11_=8.74  *p*=0.005  a,b,b,-,- | F_2,12_=7.40 *p*=0.008  a,b,b,-,- | F_3,18_=15.9 *p*<0.001  a,a,b,c,- | F_3,18_=30.6 *p*<0.001  a,a,a,b,- | F_3,16_=2.50 *p*=0.096 | F_3,18_=12.9 *p*<0.001  a,a,a,b,- | F_3,18_=3.54 *p=*0.036  ab,a,b,a,- | F_3,18_=3.36 *p*=0.042  a,a,b,ab,- | F_3,18_=3.31 *p*=0.044  ab,a,a,b,- | F_2,12_=5.26  *p*=0.023  a,b,ab,-,- | F_2,12_=12.1  *p*=0.001  a,b,c,-,- | F_2,12_=154  *p<*0.001  a,b,c,-,- | F_2,12_=4.01  *p*=0.046  a,ab,b,-,- | F_2,12_=0.72  *p*=0.51 |
| T×M | F_2,11_=0.19  *p*=0.83 | F_2,12_=18.9  *p*<0.001 | F_3,18_=2.83  *p*=0.068 | F_3,18_=2.94  *p*=0.061 | F_3,16_=0.76  *p*=0.53 | F_3,18_=1.60  *p*=0.23 | F_3,18_=1.14  *p*=0.36 | F_3,18_=1.78  *p*=0.19 | F_3,18_=1.17  *p*=0.35 | F_2,12_=0.22  *p*=0.81 | F_2,12_=0.27  *p*=0.77 | F_2,12_=0.90  *p*=0.43 | F_2,12_=0.99  *p*=0.40 | F_2,12_=0.67  *p*=0.53 |

Table S2 - Summary of two-way ANOVA, with factors treatment (T) and species (S) of all plant parameters prior to irrigation in February, March, May, end of May and June. For S, results of Fisherʼs LSD are indicated, with different letters indicating significantly different means (*p* < 0.05) for *Rumex*, *Tolpis*, *Tuberaria*, *Agrostis* and *Ornithopus*.

February

|  | F_v_/F_m pre_ | F_v_/F_m mid_ | Ψ_pre_ | Ψ_mid_ | LWC | *A* | *g*_s_ | *E* | WUE | δ^15^N | δ^13^C | %N | %C | % cover |
| --- | --- | --- | --- | --- | --- | --- | --- | --- | --- | --- | --- | --- | --- | --- |
| T | F_1,28_=0.38 *p*=0.54 | F_1,28_=1.49  *p*=0.23 | F_1,24_=0.13  *p*=0.72 | F_1,24_=0.34  *p*=0.57 | F_1,26_=1.83  *p*=0.18 | F_1,15_=3.23  *p*=0.092 | F_1,15_=0.31  *p*=0.59 | F_1,15_=1.04  *p*=0.33 | F_1,15_=2.16  *p*=0.16 | F_1,30_=3.05 *p*=0.091 | F_1,30_=4.53  *p*=0.042 | F_1,30_=0.56  *p*=0.46 | F_1,30_=0.01  *p*=0.91 | F_1,30_=0.06  *p*=0.80 |
| S | F_4,28_=10.1  *p<*0.001  a,a,a,b,b | F_4,28_=7.61  *p<*0.001  a,ab,ab,bc,c | F_3,24_=15.2  *p<*0.001  a,b,-,a,b | F_3,24_=5.96  *p=*0.003  a,a,-,b,a | F_4,26_=49.2  *p<*0.001  a,b,ac,d,c | F_2,15_=10.8  *p=*0.001  a,b,-,-,b | F_2,15_=6.65  *p=*0.009  a,b,-,-,b | F_2,15_=7.37  *p=*0.006  a,b,-,-,b | F_2,15_=2.34  *p=*0.13 | F_4,30_=53.5 *p*<0.001  a,b,c,b,c | F_4,30_=9.74  *p*<0.001  a,b,c,ab,ab | F_4,30_=27.1  *p*<0.001  a,a,a,b,c | F_4,30_=10.7  *p*<0.001  a,b,c,c,c | F_4,30_=18.2  *p*<0.001  ab,a,b,c,d |
| T×S | F_4,28_=1.43  *p=*0.25 | F_4,28_=7.14  *p*<0.001 | F_3,24_=0.39  *p*=0.76 | F_3,24_=2.64  *p*=0.072 | F_4,26_=0.74  *p*=0.58 | F_2,15_=0.25  *p*=0.78 | F_2,15_=0.14  *p*=0.87 | F_2,15_=0.42  *p*=0.67 | F_2,15_=0.69  *p*=0.52 | F_4,30_=1.46  *p*=0.24 | F_4,30_=0.50  *p*=0.73 | F_4,30_=0.37  *p*=0.83 | F_4,30_=0.38  *p*=0.82 | F_4,30_=0.13  *p*=0.97 |

March

|  | F_v_/F_m pre_ | F_v_/F_m mid_ | Ψ_pre_ | Ψ_mid_ | LWC | *A* | *g*_s_ | *E* | WUE | δ^15^N | δ^13^C | %N | %C | % cover |
| --- | --- | --- | --- | --- | --- | --- | --- | --- | --- | --- | --- | --- | --- | --- |
| T | F_1,30_=0.01 *p*=0.94 | F_1,29_=0.68  *p*=0.42 | F_1,30_=14.8  *P<*0.001 | F_1,30_=0.05  *p*=0.82 | F_1,30_=7.92  *p*=0.009 | F_1,22_=5.95  *p*=0.023 | F_1,22_=4.57  *p*=0.044 | F_1,22_=5.76  *p*=0.025 | F_1,22_=0.53  *p*=0.48 | F_1,29_=1.83 *p*=0.19 | F_1,29_=18.8 *p*<0.001 | F_1,30_=0.36 *p*=0.55 | F_1,29_=0.30 *p*=0.59 | F_1,30_=0.00 *p*=0.96 |
| S | F_4,30_=5.90  *p=*0.001  a,ab,a,bc,c | F_4,29_=8.65  *p<*0.001  a,bc,ab,d,cd | F_4,30_=12.7  *p<*0.001  a,a,a,b,c | F_4,30_=31.9  *P<*0.001  a,b,b,c,b | F_4,30_=59.7  *p<*0.001  a,b,c,d,ac | F_3,22_=7.50  *p=*0.001  a,b,b,-,b | F_3,22_=5.79  *p=*0.004  a,b,b,-,b | F_3,22_=4.48  *p=*0.013  a,b,b,-,b | F_3,22_=0.98  *p=*0.42 | F_4,29_=56.2 *p*<0.001  a,b,c,b,c | F_4,29_=13.2 *p*<0.001  a,b,b,a,a | F_4,30_=25.0 *p*<0.001  a,a,a,b,c | F_4,29_=4.81 *p*=0.004  a,b,a,b,ab | F_4,30_=20.3  *P<*0.001  a,a,a,b,c |
| T×S | F_4,30_=0.78  *p=*0.55 | F_4,29_=0.61  *p*=0.66 | F_4,30_=1.58  *p*=0.21 | F_4,30_=1.87  *p*=0.14 | F_4,30_=0.95  *p*=0.45 | F_3,22_=2.69  *p*=0.071 | F_3,22_=1.32  *p*=0.29 | F_3,22_=0.99  *p*=0.42 | F_3,22_=0.13  *p*=0.94 | F_4,29_=1.90  *p*=0.14 | F_4,29_=1.00  *p*=0.42 | F_4,30_=0.89  *p*=0.48 | F_4,29_=0.91  *p*=0.47 | F_4,30_=0.33  *p*=0.85 |

May

|  | F_v_/F_m pre_ | F_v_/F_m mid_ | Ψ_pre_ | Ψ_mid_ | LWC | *A* | *g*_s_ | *E* | WUE | δ^15^N | δ^13^C | %N | %C | % cover |
| --- | --- | --- | --- | --- | --- | --- | --- | --- | --- | --- | --- | --- | --- | --- |
| T | F_1,30_=0.05 *p*=0.83 | F_1,30_=2.44  *p*=0.13 | F_1,30_=3.38  *P=*0.076 | F_1,30_=3.38  *p*=0.076 | F_1,28_=5.11  *p*=0.032 | F_1,23_=1.15  *p*=0.29 | F_1,23_=5.05  *p*=0.035 | F_1,23_=17.4  *P<*0.001 | F_1,23_=4.73  *p*=0.040 | F_1,27_=2.99 *p*=0.095 | F_1,28_=11.9  *p*=0.002 | F_1,28_=0.00 *p*=0.95 | F_1,28_=5.63 *p*=0.025 | F_1,30_=0.07 *p*=0.79 |
| S | F_4,30_=7.42  *P<*0.001  a,a,ab,b,c | F_4,30_=8.45  *p<*0.001  a,b,a,c,bc | F_4,30_=37.4  *p<*0.001  a,b,bd,c,d | F_4,30_=27.6  *P<*0.001  a,a,b,c,b | F_4,28_=88.9  *p<*0.001  a,b,c,d,c | F_3,23_=5.73  *p=*0.004  a,bc,ab,-,c | F_3,23_=2.20  *p=*0.12 | F_3,23_=0.93  *p=*0.44 | F_3,23_=2.62  *p=*0.076 | F_4,27_=42.3  *p*<0.001  a,b,c,b,c | F_4,28_=13.2  *p*<0.001  a,b,bc,d,cd | F_4,28_=4.48  *p*=0.006  ab,b,a,a,b | F_4,28_=10.3  *p*<0.001  a,b,ac,c,c | F_4,30_=20.0  *p*<0.001  a,b,b,c,a |
| T×S | F_4,30_=0.45  *p=*0.77 | F_4,30_=0.60  *p*=0.67 | F_4,30_=1.09  *p*=0.38 | F_4,30_=0.97  *p*=0.44 | F_4,28_=0.59  *p*=0.67 | F_3,23_=0.12  *p*=0.95 | F_3,23_=1.48  *p*=0.25 | F_3,23_=1.27  *p*=0.31 | F_3,23_=1.86  *p*=0.17 | F_4,27_=1.41  *p*=0.26 | F_4,28_=0.64  *p*=0.64 | F_4,28_=0.13  *p*=0.97 | F_4,28_=0.85  *p*=0.51 | F_4,30_=0.38  *p*=0.82 |

End of May June

|  | Ψ_pre_ | Ψ_mid_ | LWC | *A* | *g*_s_ | *E* | WUE |  |  | Ψ_pre_ | Ψ_mid_ | LWC | *A* | *g*_s_ | *E* | WUE |
| --- | --- | --- | --- | --- | --- | --- | --- | --- | --- | --- | --- | --- | --- | --- | --- | --- |
| T | F_1,24_=0.03  *p*=0.87 | F_1,23_=5.24  *p*=0.032 | F_1,29_=4.82  *p*=0.036 | F_1,22_=4.21  *p*=0.052 | F_1,22_=0.23  *p*=0.63 | F_1,22_=0.00  *p*=0.98 | F_1,22_=3.06  *p*=0.094 |  | T | F_1,18_=0.31  *p*=0.58 | F_1,18_=3.24  *p*=0.089 | F_1,18_=0.48  *p*=0.50 | F_1,17_=18.9  *p<*0.001 | F_1,17_=7.20  *p*=0.016 | F_1,17_=4.14  *p*=0.058 | F_1,17_=0.05  *p*=0.83 |
| S | F_3,24_=9.17  *p<*0.001  a,b,b,-,b | F_3,23_=10.2  *p<*0.001  a,b,b,-,b | F_4,29_=176  *p<*0.001  a,b,a,c,d | F_3,22_=13.6  *p<*0.001  a,b,b,-,c | F_3,22_=5.96  *p=*0.004  a,ab,c,-,bc | F_3,22_=4.85  *p=*0.010  a,ab,b,-,b | F_3,22_=4.42  *p=*0.014  ab,a,a,-,b |  | S | F_2,18_=6.88  *p=*0.006  a,a,b,-,- | F_2,18_=18.9  *p<*0.001  a,b,c,-,- | F_2,18_=8.66  *p=*0.002  a,b,a,-,- | F_2,17_=13.5  *p<*0.001  a,b,c,-,- | F_2,17_=7.98  *p=*0.004  a,a,b,-,- | F_2,17_=4.54  *p=*0.026  ab,a,b,-,- | F_2,17_=0.79  *p=*0.47 |
| T×S | F_3,24_=2.43  *p*=0.090 | F_3,23_=0.90  *p*=0.46 | F_4,29_=1.53  *p*=0.22 | F_3,22_=0.26  *p*=0.86 | F_3,22_=1.94  *p*=0.15 | F_3,22_=1.28  *p*=0.31 | F_3,22_=0.84  *p*=0.49 |  | T×S | F_2,18_=0.09  *p*=0.92 | F_2,18_=0.22  *p*=0.81 | F_2,18_=2.01  *p*=0.16 | F_2,17_=0.93  *p*=0.41 | F_2,17_=0.31  *p*=0.74 | F_2,17_=0.87  *p*=0.44 | F_2,17_=0.13  *p*=0.88 |
